# The Medical Schools of the United Kingdom

**Published:** 1913-09-06

**Authors:** 


### THE MEDICAL STUDENT AND HIS WORK.

### THE MEDICAL SCHOOLS OF THE UNITED KINGDOM.

### LONDON.

*St. Bartholomew's Hospital Medical School.*—St. Bartholomew's is the oldest and second largest hospital in London. The Medical School is complete in every department, and provides lectures and practical laboratory teaching in the preliminary sciences and intermediate subjects, as well as in the purely professional and clinical parts of the curriculum. Scholarships and prizes to the total value of £1,000 are awarded annually.

*Charing Cross Hospital.*—This hospital contains 200 beds, and has attached to it a Medical School. Classes for University, Conjoint, and Fellowship courses are held, and there are special classes for the practical work required for the D.P.H. course. Total fees, including students' club: For general students—Sessional payments: Entrance fee 10 guineas and 25 guineas annually for University students, 22 guineas annually for Conjoint Board students, so long as the student remains in the school. Special arrangements are made to permit of fees being paid in advance. The usual resident and students' appointments are open to students, and several valuable prizes are awarded annually by the school. Full information respecting the Medical School, and the course of study recommended, may be obtained on application to the Dean, Dr. William Hunter, F.R.C.P., Medical College, Charing Cross Hospital, London, W.C.

*St. George's Hospital.*—The winter session commences on October 1, when Sir Wm. Osler, Bart., Regius Professor of Medicine, Oxford University, will deliver the Inaugural Address, taking for his subject "Examinations, Examiners, Examinees!" Students can enter at any time. Special courses are given, in which the requirements of University and other examinations receive careful attention. The hospital contains 350 beds, and has a convalescent hospital of 100 beds at Wimbledon. The usual resident and students' appointments are open to students. Scholarships and endowed prizes, amounting to £463, are awarded every year. The entire teaching and laboratories are now devoted to purely clinical subjects, as in other Universities, to the great advantage of students in their fourth and fifth years of study. Arrangements have been made with the University of London for students who enter St. George's during the first, second, or third year of the curriculum to carry out the necessary courses of instruction at either University College or King's College. Students have therefore the advantages of the lectures and practical classes of these Colleges of the University during the preliminary and intermediate portions of their studies, and then complete their course in a school entirely devoted to medicine, surgery, and the study of disease. The St. George's Hospital Club has its athletic ground of 13½ acres on the Atkinson-Morley Hospital estate at Wimbledon. It provides a football and hockey ground and six tennis courts. Further particulars may be obtained on application to the Dean of the Medical School, Dr. R. Salusbury Trevor.

*Guy's Hospital.*—This hospital contains 618 beds, and has a large Medical School. There are special departments, and a large number of student and resident appointments available. Special classes are held for the London University, Oxford, Cambridge, and Fellowship courses. Scholarships and prizes to the amount of £800 are awarded annually. For further particulars application should be made to the Dean of the Medical School, Dr. H. C. Cameron.

*King's College Hospital.*—King's College Hospital, which contains 224 beds, is situated at Denmark Hill, S.E., and will be opened on October 1 next. It will be on a much larger scale than the former institution, and will possess every modern requirement for both patients and students. The teaching of the preliminary subjects, including chemistry, physics, biology, anatomy, and physiology, is given at King's College in the Strand, and students entering now will by the time they are sufficiently advanced to study in the wards to do so in the new hospital. The composition fee is—for advanced medical students—80 guineas; for the whole University or Conjoint course, 150 guineas. Full particulars and prospectuses can be obtained by applying to the Dean of the Hospital, Dr. H. Willoughby Lyle, or to the Secretary of the Medical School, Clifton Kelway, Esq.

*St. Mary's Hospital Medical School.*—St. Mary's Hospital contains at present 301 beds. Systematic clinical teaching is given daily in the wards and out-patients' department and in the various special departments of the hospital. The Medical School provides for the entire curriculum, and contains well-organised departments for preliminary scientific subjects. The composition fee for the whole curriculum is £140, or 60 guineas for the clinical curriculum only.

*The Middlesex Hospital.*—The hospital and Medical School are situated in Mortimer Street, and only a few minutes' walk from Goodge Street Station (Hampstead and Charing Cross Tube), Oxford Circus Stations (Bakerloo and Central London Tubes), and Portland Road Station (Metropolitan Railway).

The hospital contains 440 beds, including special wards for cancer cases, maternity and gynæcological cases, and for diseases of the skin and eye.

The cancer wing (containing ninety beds) and special investigation laboratories offer unrivalled opportunities for the study of cancer, both in its clinical and pathological aspects.

In the electro-therapeutical department students obtain instruction in the treatment of lupus and cancer by the *x*-ray method.

There is a Residential College for a limited number of students.

Six house physicians, eight house surgeons, two obstetric and gynæcological surgeons, two casualty medical officers, two casualty surgical officers, and two resident officers to the special departments are appointed annually, without extra fee of any kind. Non-resident qualified clinical assistants are appointed to assist in the various out-patient departments.

Three Entrance Scholarships, value £100, £50, and £25, and a University Scholarship, value £50 (for Oxford and Cambridge students), are awarded annually in September. The successful candidates are required to become general students of the school. Numerous scholarships, medals, and prizes, to the value of over £1,000, are open for competition among students of the school.

All students join the Amalgamated Students' Club, which includes the following:—The Medical Society, the Common Room Society, the Cricket Club, the Football

Clubs, the Athletic Clubs, the Rowing Club, the Musical Society, the Chess Club, the Lawn-tennis Club, and the Hockey Club. The athletic ground, which is eight acres in extent, is situated within easy access of the hospital—at Park Royal. There is a gymnasium within the precincts of the hospital.

The winter session, 1913-1914, will open on Wednesday, October 1, at 3 P.M. The Introductory Address will be delivered by W. Sampson Handley, Esq., M.D., M.S., F.R.C.S., after which the prizes will be distributed by Sir Squire Bancroft.

For prospectus and full particulars application should be made to the Dean of the Medical School.

*The London Hospital Medical College.*—The London Hospital is the largest institution of its kind in England, and contains 922 beds. Being situated in the East End, it ministers to a very large and poor district. The total number of patients during 1912 was 243,834, made up of 16,827 in-patients and 227,007 out-patients. During the year 5,256 major operations were performed in the theatre, while the number of operations under anæsthesia was 21,555. Five entrance scholarships are offered annually, including the Price Scholarship in science, of £100; the Epsom Scholarship; and the entrance scholarships in science, anatomy and physiology, and arts. The annual inclusive fee is 30 guineas. Appointments: Registrars, resident accoucheurs, house physicians, house surgeons, etc. One hundred and forty-one appointments are made annually. All appointments are free to full students, and all resident appointments have board free. Full information may be obtained from the Dean of the Medical College. A Hostel for students is provided on the hospital ground. The Clubs Union Athletic Ground is within easy reach of the hospital.

*University College Hospital Medical School* comprises the departments of medicine and clinical medicine, surgery and clinical surgery, midwifery and gynæcology, pathology and morbid anatomy and clinical pathology, bacteriology, mental physiology and mental diseases, dental surgery, practical pharmacy, and other departments for the study of special diseases such as those of the eye, skin, ear, and throat, and for instruction in the use of anæsthetics, and in electro-therapeutics and the application of the *x*-rays. Scholarships and exhibitions to the value of about £800 are offered for competition every year. Composition fees for the courses required by the University of London: For the final M.B., B.S. course, 80 guineas, or in two instalments of 50 and 32 guineas; for the medical education required by the Examining Board in England and the Society of Apothecaries, for the course required for the third examination, 80 guineas, or in two instalments of 50 and 32 guineas.

*University of London, University College* (Faculty of Medical Sciences): University Centre for Preliminary and Intermediate Medical Studies.—The College Faculty of Medical Sciences comprises the departments of physics, chemistry, botany, and zoology (the preliminary medical sciences), also the departments of anatomy, physiology, and pharmacology (the intermediate medical sciences), and the departments of hygiene and public health, and pathological chemistry (post-graduate study). Composition Fees: For the courses required by the University of London: (1) For the first medical course, 26 guineas, entitling to one attendance. (2) For the second medical course, 58 guineas if paid in one sum; 63 guineas if paid in two instalments of 32 guineas and 31 guineas respectively. This fee entitles to attendance on anatomy and physiology during three years, and to one attendance on organic and applied chemistry, pharmacology and materia medica, and to the privileges of the Union Society (including the use of the gymnasium and the athletic ground at Perivale) for two sessions. For the medical education required by the Examination Board in England and the Society of Apothecaries: First examination, Parts I., II., III., 21 guineas, entitling to one attendance and to the privileges of the Union Society for one session. First examination, Part IV., and second examination, 58 guineas, if paid in one sum, and 63 guineas if paid in two instalments of 32 guineas and 31 guineas respectively. This fee entitles to attendance during three years (including one attendance on each practical course in physiology) in all subjects but practical pharmacy, the fee for which is three guineas, and to the privileges of the Union Society (including the use of the gymnasium and the athletic ground at Perivale) for two sessions.

*Westminster Hospital Medical School.*—Westminster Hospital was the first institution of its kind in London which was founded by the voluntary contributions of the public. There is accommodation for upwards of 200 in-patients. There is a Students' Clubs Union, the subscription for membership of which is included in the general fees. The annual composition fee is 25 guineas. Special terms are given to sons of medical men. Five Entrance Scholarships are offered for competition amongst students entering in October and three for those entering in May.

*London (Royal Free Hospital) School of Medicine for Women.*—The Royal Free Hospital is in Gray's Inn Road, and the London School of Medicine for Women is in Hunter Street, Brunswick Square, W.C. There are 165 beds in the hospital, all of which are available for clinical teaching. There are numerous scholarships and prizes offered annually in connection with the school, among which may be mentioned the Isabel Thorne Scholarship of £30, the St. Dunstan's Medical Exhibition of £60 a year for three years, extendible to five years, the Mabel Sharman-Crawford Scholarship of £20 a year for four years, and the Agnes Guthrie Bursary for dental students of £60. Various resident appointments at the Royal Free Hospital are available for students of the school after qualification.

*The Hospital for Women, Soho.*—In connection with the out-patient department there has been for some years a well-organised clinical department. To meet the want increasingly felt by medical practitioners of a more intimate knowledge of the diseases peculiar to women, and of more extended opportunities for examining gynæcological cases, clinical assistants are appointed to the honorary medical officers to out-patients. The appointments are open to qualified medical men and women.

The hospital contains sixty-seven beds. In the out-patient department there are 4,000 new cases during the year, the total number of out-patient attendances being from 14,000 to 16,000. This large number affords exceptional opportunities for seeing and studying most of the varieties of the diseases of women. Clinical assistants are entitled to receive notice of all operations performed within the hospital upon giving their names and addresses to the hall porter, and every facility is afforded them by the honorary medical officers in the out-patient department of examining patients and obtaining experience in diagnosis and treatment.

The number of clinical assistants appointed to each gynæcologist is so far as possible limited to two, though occasionally a third may be appointed. The clinical assistants attend on two afternoons a week, and it is advisable that they should take out a three months' course, but to meet the convenience of medical practitioners it is permitted to attend for a shorter period, and on one or more days a week if it can be arranged.

A fee is charged at the rate of two guineas a month, which becomes due on joining. A certificate is given at the end of a three months' course.

The out-patients are seen daily at 1.30, except on Saturdays, when they are seen at 9.30.

Any further information can be obtained by writing to or calling on the Secretary at the hospital.

### PROVINCIAL SCHOOLS.

*The University of Birmingham (Faculty of Medicine).*—In order to obtain the degrees of M.B. and Ch.B. five examinations have to be passed. The first is in chemistry, physics, and elementary biology; the second in anatomy and physiology; the third in pathology, bacteriology, and materia medica and pharmacy; the fourth in forensic medicine, toxicology, public health, and therapeutics; and the fifth, or final, in medicine, surgery, midwifery, diseases of women, mental diseases, and ophthalmology. The Univsity also grants degrees and a diploma in dental surgery, and a diploma (D.P.H.) and a degree in Public Health (B.Sc.). The General Hospital and the Queen's Hospital, containing between them over 500 beds, are amalgamated for the purpose of clinical instruction, which is carried on under the direction of the University Clinical Board. Medical students also have free access to clinical instruction at the Eye, Ear and Throat, Orthopædic and Spinal, Women's, and Children's Hospitals, which are associated with the University. The composition fee for the whole course of lectures and instruction at the University is £85, besides which there is a composition fee for attendance on the medical and surgical practice and the clinical lectures at both the General and Queen's Hospitals of £42, making a total of £127. Dean: Professor Peter Thompson, M.D. The winter session opens on Tuesday, October 7, 1913.

*University of Bristol*—The University grants the conjoined degrees of M.B., Ch.B., the degree of Doctor of Medicine (M.D.), Master of Surgery (Ch.M.), Bachelor of Dental Surgery (B.D.S.) and Master of Dental Surgery (M.D.S.); also diplomas in Public Health (D.P.H.), in Dental Surgery (L.D.S.), and in Veterinary State Medicine (D.V.S.M.). For the conjoined degrees of M.B., Ch.B., the curriculum occupies five years and a half. The University lectures and laboratory courses are attended in the large and well-equipped new wing of the University. Hospital practice and clinical instruction are provided in the allied hospitals, which together afford upwards of six hundred beds and a very large external midwifery department. Three years at least must be spent in the University, and two of them subsequently to passing the second examination. Women are admitted to all degrees and diplomas and to the courses of study necessary for them. The University Hall of Residence is situated at Clifton, within a short distance of the University. Particulars of residence may be obtained from Miss M. C. Staveley, M.A., at the University. The inclusive fee for the M.B., Ch.B. curriculum is 140 guineas. The winter session opens on October 1.

*University College, Cardiff.*—Since it was founded in 1883, University College, Cardiff, has prepared students for the Preliminary Scientific examination of the University of London, and for the corresponding examinations of other licensing bodies. Chairs of Anatomy, Physiology, and Pathology and Bacteriology, and Lectureships in Materia Medica and Pharmacy and Histology and Embryology have been established, making it possible to spend at least three out of the five years of the medical curriculum at this school. Arrangements have been made with the managing committee of the Cardiff Infirmary to give students of the college the privilege of attending this hospital, which contains 196 beds. The composition fee for the three years' course of study for the Preliminary Scientific and Intermediate Examination for the London M.B. is £63.

*The University of Leeds (School of Medicine).*—This University grants the degrees of M.B. and Ch.B., M.D. and Ch.M., and in association with the Universities of Liverpool, Manchester, and Sheffield, holds a Matriculation examination. Clinical instruction is given in the General Infirmary, which contains 520 beds, and is amply provided with special departments. Students of the University also have access for instruction to the Leeds Public Dispensary, the City Fever Hospitals, the Hospital for Women and Children, the Leeds Maternity Hospital, and the West Riding Asylum at Wakefield. Diplomas in Public Health and Psychological Medicine are also granted.

*University of Durham.*—For students who intend to graduate at the Durham University it is necessary that one of the five years of professional education should be spent in attendance at the College of Medicine, Newcastle-upon-Tyne. During the year so spent the student must attend lectures at the College and the medical and surgical practice and clinical lectures at the Royal Victoria Infirmary, which contains over 400 beds. No residence at Durham is necessary, the Medical Faculty being in Newcastle-upon-Tyne. The remaining four years of the curriculum may be spent at Newcastle-upon-Tyne or at one or more of the recognised medical schools. The composition fee for the complete course of lectures is 72 guineas, and for the medical and surgical practice of the hospital 35 guineas. There are four professional examinations for the M.B., B.S. degrees. Fees, £25. Address, Dr. Robert Howden, The College of Medicine, Newcastle-upon-Tyne.

*The Victoria University of Manchester (Faculty of Medicine).*—The medical department of the University is particularly well provided with facilities for instruction in all the courses for degrees and the professional qualification and for teaching the preliminary subjects—namely, anatomy, physiology, pathology, materia medica, biology, physics, and chemistry. Clinical instruction is provided at the Manchester Royal Infirmary, which contains 592 beds, and also has large out-patient and casualty departments. Associated with the Infirmary are a Convalescent Hospital at Cheadle, containing 136 beds, and the Royal Lunatic Hospital, accommodating 430 patients. Clinical instruction in gynæcology is also given at St. Mary's Hospitals and other hospitals.

*The University of Liverpool (Faculty of Medicine).*—The University grants degrees in Medicine (M.B.; M.D.), in Surgery (Ch.B.; Ch.M.), and in Hygiene (M.H.). Candidates for degrees are required to pass the Matriculation examination or an equivalent. Students may study in the University for the degrees or for the examinations of other licensing bodies. The laboratories are modern and very well equipped, and the facilities for instruction are most complete. Courses of instruction in tropical diseases can be obtained at the Liverpool School of Tropical Medicine. Fellowships, scholarships, and prizes of over £1,000 are awarded annually, in addition to entrance scholarships. The Clinical School of the

University now consists of four general hospitals—the Royal Infirmary, the David Lewis Northern Hospital, the Royal Southern Hospital, and the Stanley Hospital; and of five special hospitals—the Eye and Ear Infirmary, the Hospital for Women, the Infirmary for Children, St. Paul's Eye Hospital, and St. George's Hospital for Skin Diseases. These hospitals contain in all a total of 1,127 beds. The organisation of these hospitals to form one teaching institution provides the medical student and the medical practitioner with a field for clinical education and study which is unrivalled in extent in the United Kingdom. The University also grants a diploma and degrees in dental surgery, a diploma in public health, a diploma in tropical medicine, and a diploma in ophthalmic surgery. The Dental Hospital was opened in January 1910, and its equipment for teaching purposes is complete.

*The University of Sheffield (Faculty of Medicine.).*—The degrees of the University, M.B., Ch.B., M.D., Ch.M., and the diploma in public health are open to men and women alike. A candidate for the degrees of M.B., Ch.B., must produce certificates that he will have attained the age of 21 years on the day of graduation; that he has pursued the courses of study required by the University Regulations during a period of not less than five years subsequently to the date of his registration as a medical student by the General Medical Council, three of such years at least having been passed in the University, one at least being subsequent to the passing of the first examination. The University is within easy reach of the various hospitals with which it is connected for clinical purposes. The composition fee for University lectures and practical classes is £80, payable in three instalments. Composition fee for medical and surgical hospital practice (except pharmacy) for the full period required by the University and the various Examining Boards, £49 17s. 6d., payable in three instalments. The University of Sheffield offers many valuable scholarships and prizes to students in the faculty of medicine.

### SCOTLAND.

*The Carnegie Trust and the Payment of Class Fees.*—This is a fund to provide under certain regulations eligible students with all or a portion of the class fees of the curriculum. Applicants must be over sixteen years of age, of Scottish birth or extraction, and must have obtained the Leaving Certificate of the Scotch Education Department or recognised equivalent. Forms of application are to be obtained from the Secretary, Carnegie Trust for the Universities of Scotland, 22 Hanover Street, Edinburgh.

*University of Edinburgh.*—Four degrees in medicine and surgery are conferred by the University, namely, Bachelor of Medicine (M.B.), Bachelor of Surgery (Ch.B.), Doctor of Medicine (M.D.), and Master of Surgery (Ch.M.). The degree of Ch.B. is not conferred on any person who does not at the same time obtain the degree of M.B., and the degree of M.B. is not conferred on any person who does not at the same time obtain the degree of Ch.B. A University diploma is granted in Tropical Medicine and Hygiene (D.T.M. and H.), and a University certificate is granted in Diseases of Tropical Climates. There has also been recently formulated regulations for a Diploma in Psychiatry.

To obtain the Edinburgh medical degree it is necessary to pass the preliminary examination or its recognised equivalent, and to pass four professional examinations. The first is in botany, zoology, physics, and chemistry; the second in anatomy and physiology; the third in pathology, materia medica, and therapeutics; and the final in surgery, clinical surgery, medicine, clinical medicine, midwifery, clinical gynæcology, forensic medicine, and public health. In order to obtain the M.B., Ch.B. degrees, it is necessary to spend at least two of the five years of medical study in the University of Edinburgh. Clinical instruction is given in the Royal Infirmary, which contains nearly 900 beds; Royal Hospital for Sick Children, with 120 beds; Maternity Hospital, with 40 beds available for clinical instruction; City Fever Hospital, with 600 beds, and the Royal Morningside Asylum, with 500 beds. The total number of beds available for the clinical instruction of students of the University is 2,160. The fees for the four professional examinations amount to £23 2s. Communications should be addressed to the Dean of the Medical Faculty, University New Buildings, Edinburgh, and letters respecting the preliminary examination to Mr. James Dowie, Clerk of Senatus, The University, Edinburgh.

*School of Medicine of the Royal Colleges, Edinburgh.*—This School of Medicine is constituted by an association of lecturers who lecture in several buildings near the Royal Infirmary. The lecturers are recognised or licensed by the Royal Colleges of Surgeons and Physicians, and also by the University. The teaching is similar to that of the Scottish Universities, and the students receive similar certificates at the close of each session. The lectures qualify for the University of Edinburgh and other Universities; the Royal Colleges of Physicians and Surgeons of Edinburgh, London, and Dublin; the Faculty of Physicians and Surgeons of Glasgow, and the other Medical Boards. The whole education required for graduation at the University of London may be taken in this school. The anatomy rooms open on October 1, and the lectures begin on October 7. The facilities for clinical instruction are similar to those provided for the University, and there are special classes for women. Full particulars and copy of the calendar of the school may be had gratis from the Dean of the School, 11 Bristo Place, Edinburgh.

*School of Medicine for Women, Edinburgh.*—Precisely the same facilities for medical study are given as to male students in the School of Medicine, Edinburgh. Regulations, fees, and arrangements are the same. The lecturers are recognised by the University of Edinburgh as giving courses admitting to the degrees of M.B., Ch.B. Clinical instruction is given in the Royal Infirmary and other hospitals mentioned above. The benefit of the Carnegie Trust is open to students of the college. Most of the classes are held at Surgeons' Hall. A sitting-room is provided for the students. The office of the Dean of the School is also at Surgeons' Hall. Applications for prospectuses should be addressed to Miss Keith, Secretary, School of Medicine for Women, Surgeons' Hall, Edinburgh.

*University of Glasgow.*—The whole course of study for the M.B., Ch.B., can be passed in the Medical School of the University. The regulations are similar to those given above for Edinburgh. Clinical instruction is given at the Western Infirmary, which contains about 600 beds, at the Royal Infirmary, which contains over 600 beds, and also at the Lunatic Asylum, the Eye Infirmary, the Maternity Hospital, the City Fever Hospitals, and various other hospitals. The cost of the course, including matriculation, fees for classes, for hospital attendance, and for professional examinations, amounts to about £150.

*Queen Margaret College, Glasgow (the Women's Department of the University).*—This is an integral part of the University of Glasgow. The courses, regulations, and fees are the same as for men. The instruction is given by University professors and lecturers appointed by the

University Court, partly in mixed and partly in separate classes. The College provides a separate building, with class-rooms, laboratories, library, and other teaching appliances. The women have all the rights and privileges of University students. They do their clinical work in the Royal Infirmary, and in other hospitals.

*Anderson's College Medical School, Glasgow, W.*—The college, at which a full medical curriculum can be obtained, is situated in Dumbarton Road, adjoining the Western Infirmary and the University. Degrees and diplomas: Certificates of attendance on the lectures are accepted by the Universities of London and Durham, by the Universities of Glasgow, Edinburgh, etc., under conditions stated in the calendars, and by all the Royal Colleges and licensing boards in the United Kingdom. The public health course qualifies for the Scottish Licensing Board, the Irish Colleges, and the Universities of Oxford, Cambridge, London, etc. The Carnegie Trust extends its benefactions to students of Anderson's College. Particulars may be obtained from Sir W. S. McCormick, LL.D., the Carnegie Trust Offices, Edinburgh. A calendar will be sent on receipt of a postcard by the Secretary to the Medical Faculty, Anderson's College Medical School, Glasgow, W.

*St. Mungo's College: the Medical School of the Glasgow Royal Infirmary.*—The college buildings are situated within the grounds of the infirmary, and are provided with class-room and laboratory accommodation for several hundred students. Students receive their clinical instruction in the wards of the infirmary, which contains 600 beds, and in the Glasgow Maternity and Women's Hospital. The College provides a complete medical curriculum, and the classes qualify for the diplomas of the English, Scottish, and Irish Conjoint Boards, and under certain conditions for the various Universities. Students who have fulfilled the conditions of the Carnegie Trust are eligible for the benefits of this Trust during the whole course of their studies at St. Mungo's College. The classes at St. Mungo's College are open to male and female students equally. Students entering for the dental diploma can take out almost all their classes at St. Mungo's College. There is a special department of public health, attendance on which qualifies for the Conjoint Boards and the Universities of Oxford and Cambridge. The inclusive fees for the whole medical curriculum amount to about £70.

*University of Aberdeen.*—The regulations are practically the same as those of the other Scottish Universities. Clinical instruction is obtained at the Royal Infirmary, which contains 250 beds, in the Sick Children's Hospital, and several other institutions, including the Royal Lunatic Asylum and the City Fever Hospital. At least two of the five years of study and at least six of twelve specified subjects must be spent or taken in the University. The remaining three years may be spent and the remaining subjects taken in any University of the United Kingdom, or in any Indian, Colonial, or foreign University, recognised for the purpose by the University Court, or in recognised Medical Schools or under recognised teachers. There are four professional examinations, and the cost of Matriculation, class and degree fees for the whole curriculum is about £160. Address, Mr. D. R. Thom, M.A., Secretary, The University, Aberdeen.

*University of St. Andrews.*—Medical students may attend for the first two years of study either in St. Andrews or in Dundee. The remainder of the course must be taken in Dundee, and full particulars respecting the curriculum and examinations may be obtained from Professor Kynoch, Dean of the Faculty of Medicine, the Medical School, Dundee. Clinical instruction is given at the Dundee Royal Infirmary, which has 400 beds, at the Dundee Eye Institution, the King's Cross Fever Hospital, and the Dundee District Asylum.

*Western Medical School, Glasgow.*—This school is situated in University Avenue, opposite to the principal gate of the University. Lectures and demonstrations are given in the usual winter and summer sessions on chemistry, anatomy, surgery, medicine, midwifery, and gynæcology, ophthalmology, and dermatology. Some of the classes qualify for graduation and for Scottish diplomas. The usual class fee is £2 2s. There is no Matriculation fee. Further information may be obtained from the Secretary.

### IRELAND.

*University of Dublin (Trinity College).*—All students in the School of Physics must pass a matriculation examination. This may be either the public entrance and a term examination of Trinity College or a special medical preliminary, or, for extern students, an examination recognised by the General Medical Council. No student can be admitted for the winter course after November 17. Candidates for the degrees in Medicine (M.B.), Surgery (B.Ch.), and Midwifery (B.A.O.) must be of B.A. standing and must be for at least five academic years on the books of the Medical School, reckoned from the date of Matriculation. The Arts course may be concurrent with the Medical course, and the B.A. degree need not be taken before the final medical examinations, but the Medical degrees are not conferred without the Arts degree.

*Royal College of Surgeons in Ireland (the Schools of Surgery).*—These schools are attached by charter to the Royal College of Surgeons in Ireland. They are carried on within the college buildings, and are specially subject to the supervision and control of the Council, which is empowered to appoint and remove the professors, and to regulate the methods of teaching pursued. The buildings have been reconstructed, the capacity of the dissecting-room nearly trebled, and special pathological, bacteriological, public health, chemical, and pharmaceutical laboratories fitted with the most approved appliances, in order that students may have the advantage of the most modern methods of instruction. There are special rooms set apart for lady students. The medical students' guide will be forwarded post free on application to the Registrar, Royal College of Surgeons, Dublin.

*The Queen's University of Belfast.*—This University provides all the classes required for a complete medical curriculum. The University contains laboratories in connection with the departments of biology, chemistry, physiology, pathology, anatomy, physics and materia medica. Women are eligible as students. Clinical instruction is given at the Royal Victoria Hospital, which was rebuilt a few years ago and has 300 beds, and at the Mater Infirmorum Hospital, which has 150 beds. Other hospitals open to the students of the University are: The Maternity Hospital, the Ulster Hospital for Women and Children, the Hospital for Sick Children, the Ophthalmic Hospital, the Benn Ulster Eye, Ear, and Throat Hospital, the Union Infirmary and Fever Hospital, the Fever Hospital, Purdysburn, and the District Lunatic Asylum, the Samaritan Hospital, the Forster Green Hospital for Consumption and Chest Diseases, the Belfast and Ulster Hospital for Diseases of the Skin. The cost of the curriculum intended for students proceeding to the degrees of the Queen's University of Belfast is, approximately, £105. This includes examination fees and a perpetual ticket for attendance at the Royal Victoria Hospital or the Mater Infirmorum Hospital, but not fees for the special hospitals. The course for the Conjoint Board costs about the same amount. A pamphlet containing full information regarding the new regulations for courses, fees, etc., can be had free of cost on application to the Secretary, Queen's University, Belfast.

*University College, Cork.*—Students can attend courses which meet the requirements of the National University of Ireland, the Conjoint Boards of London, Edinburgh, or Dublin, and other examining bodies. Clinical instruction is given at the North and South Infirmaries, each of which contains 100 beds, at the District Hospital, which contains 1,200 beds, and at other hospitals. The college is a constituent college of the National University of Ireland, and holds its own examinations in all subjects required for the degrees of that University. Twelve Entrance Scholarships, varying from £40 to £20, which may be held in any Faculty, are offered for competition. There are, in addition, twelve scholarships of about £30 each offered for competition to medical students of the second, third, fourth, and fifth years. The Blayney Scholarship, of about £32, is awarded to the candidate who, being of the prescribed standing, obtains highest marks with honours at the National University examination for the M.B., B.Ch., B.A.O. degrees, taken as a whole. Further information can be had on application to the Registrar, University College, Cork.

*University College, Galway.*—In addition to various laboratories, a dissecting-room, and anatomical lecture theatre, the College contains a large natural history and geological museum, as well as an extensive library for the students' use. New chemical and pathological laboratories are in course of construction. There are eight junior scholarships in medicine of the annual value of £25 each. Scholarship examinations are held at the beginning of each session. Clinical instruction is given in the Galway Hospital and in the Galway Union and Fever Hospitals. Full information as to courses of lectures, scholarships, and class fees can be obtained from the Registrar, University College, Galway.

*The Medical School of the Catholic University, Ireland, Dublin.*—Founded in 1855, it is now the largest medical school in Ireland. It prepares students specially for the examinations of the Royal University and the Conjoint Colleges of Ireland and Edinburgh, but its lectures and practical courses are recognised by all the licensing bodies in Great Britain and Ireland. In addition to the ordinary medical examinations it prepares students for the D.P.H. and for the various higher University examinations in pathology, physiology, chemistry, etc. Six exhibitions and numerous gold and silver medals are offered annually for competition.

